# The Global Monkeypox (Mpox) Outbreak: A Comprehensive Review

**DOI:** 10.3390/vaccines11061093

**Published:** 2023-06-12

**Authors:** Shriyansh Srivastava, Sachin Kumar, Shagun Jain, Aroop Mohanty, Neeraj Thapa, Prabhat Poudel, Krishna Bhusal, Zahraa Haleem Al-qaim, Joshuan J. Barboza, Bijaya Kumar Padhi, Ranjit Sah

**Affiliations:** 1 Department of Pharmacology, Delhi Pharmaceutical Sciences and Research University (DPSRU), Sector 3 Pushp Vihar, New Delhi 110017, India; sachinsodan@gmail.com (S.K.); shagunjain511@gmail.com (S.J.); 2 Department of Pharmacy, School of Medical and Allied Sciences, Galgotias University, Greater Noida 203201, India; 3 Department of Clinical Microbiology, All India Institute of Medical Sciences, Gorakhpur 273008, India; aroopmohanty7785@yahoo.com; 4 Nepal Medical College, Jorpati, Kathmandu 44600, Nepal; thapaneeraj16@gmail.com (N.T.); theprabhatpoudel@gmail.com (P.P.); 5 Lumbini Medical College, Tansen-11, Pravas, Palpa 32500, Nepal; krishnabhusal1994@gmail.com; 6 Department of Anesthesia Techniques, Al-Mustaqbal University College, Hilla 51001, Iraq; zahraahaleem@uomus.edu.iq; 7 Escuela de Medicina, Universidad César Vallejo, Trujillo 13007, Peru; 8 Department of Community Medicine and School of Public Health, Postgraduate Institute of Medical Education and Research, Chandigarh 160012, India; bkpadhi@gmail.com; 9 Department of Microbiology, Tribhuvan University Teaching Hospital, Institute of Medicine, Kathmandu 44600, Nepal; 10 Department of Microbiology, Dr. D. Y. Patil Medical College, Hospital and Research Centre, Dr. D. Y. Patil Vidyapeeth, Pune 411018, India; 11 Department of Public Health Dentistry, Dr. D.Y. Patil Dental College and Hospital, Dr. D.Y. Patil Vidyapeeth, Pune 411018, India

**Keywords:** monkeypox virus, orthopoxvirus, pathophysiology, genetic clade, vaccines

## Abstract

Monkeypox (Mpox) is a contagious illness that is caused by the monkeypox virus, which is part of the same family of viruses as variola, vaccinia, and cowpox. It was first detected in the Democratic Republic of the Congo in 1970 and has since caused sporadic cases and outbreaks in a few countries in West and Central Africa. In July 2022, the World Health Organization (WHO) declared a public-health emergency of international concern due to the unprecedented global spread of the disease. Despite breakthroughs in medical treatments, vaccines, and diagnostics, diseases like monkeypox still cause death and suffering around the world and have a heavy economic impact. The 85,189 reported cases of Mpox as of 29 January 2023 have raised alarm bells. Vaccines for the vaccinia virus can protect against monkeypox, but these immunizations were stopped after smallpox was eradicated. There are, however, treatments available once the illness has taken hold. During the 2022 outbreak, most cases occurred among men who had sex with men, and there was a range of 7–10 days between exposure and the onset of symptoms. Three vaccines are currently used against the Monkeypox virus. Two of these vaccines were initially developed for smallpox, and the third is specifically designed for biological-terrorism protection. The first vaccine is an attenuated, nonreplicating smallpox vaccine that can also be used for immunocompromised individuals, marketed under different names in different regions. The second vaccine, ACAM2000, is a recombinant second-generation vaccine initially developed for smallpox. It is recommended for use in preventing monkeypox infection but is not recommended for individuals with certain health conditions or during pregnancy. The third vaccine, LC16m8, is a licensed attenuated smallpox vaccine designed to lack the B5R envelope-protein gene to reduce neurotoxicity. It generates neutralizing antibodies to multiple poxviruses and broad T-cell responses. The immune response takes 14 days after the second dose of the first two vaccines and 4 weeks after the ACAM2000 dose for maximal immunity development. The efficacy of these vaccines in the current outbreak of monkeypox is uncertain. Adverse events have been reported, and a next generation of safer and specific vaccines is needed. Although some experts claim that developing vaccines with a large spectrum of specificity can be advantageous, epitope-focused immunogens are often more effective in enhancing neutralization.

## 1. Introduction

In recent years, there has been cause for concern due to a rapid and unprecedented pandemic of Mpox infections in several countries all over the world. Rodents and primates are the hosts for Mpox, a zoonosis (a virus transmitted from animals to people) with symptoms like smallpox but less severe. When the virus was initially identified in monkeys in a Danish laboratory in 1958, the term “monkeypox” was coined [1]. The etiological agent of monkeypox, a zoonotic illness that can be transmitted to humans, is the monkeypox virus, which is a member of the Orthopoxvirus genus that was originally mainly present in Central and West Africa [2,3]. There have been numerous reports of human Mpox from various countries that are not typically affected since May 2022. With the large number of proven cases and stories of the virus passing between people and within a population, it has become a cause for concern all over the world. The large number of people confirmed to have Mpox around the world currently stands at over 85,189, and it is rapidly increasing in more than 110 nations. The WHO declared it a Public Health Emergency of International Concern on 23 July 2022 in order to alert the world of the danger it poses [4]. Since smallpox was eradicated in 1980, the Mpox virus has been found to be the most prevalent orthopoxvirus impacting people. It produces a sickness that is identical to smallpox in humans. The signs and symptoms of the present Mpox epidemic differ from those in the past, even though the virus was first identified many years ago. Traditionally, the only genital sores associated with human Mpox were ones that were all the same in appearance—pustular eruptions [5,6]. The present monkeypox outbreak, however, is differentiated by genital rashes. Additionally, the vaginal rash typically comes before the widespread pustular rash in non-endemic regions outside of Africa [6,7,8,9]. An initial infection in the genital region can cause a localized rash and, in rare instances, a subsequent widespread illness. Additionally, skin lesions and prodromal symptoms are not significantly linked, and systemic symptoms are only present in around 50% of patients [10]. The clinical syndrome is characterized by lymphadenopathy, rash, and fever. Pneumonitis, encephalitis, sight-threatening keratitis, and subsequent bacterial infections are some of the possible side effects of Mpox [11]. Skin rashes can present themselves at varying times, so medical professionals and scientists should be cognizant of this new reality, which is distinct from what has been observed in the past. Many studies have shown that the way a person’s immune system works is closely linked to how their disease starts and worsens when they have a virus infection. Immune escape is a common thing in orthopoxvirus infections and plays a significant part in how they spread [12,13]. Immunological patterns may serve as potential markers of disease progression and treatment targets for Mpox. The association between intense Mpox disease and immunological response is becoming more apparent, prompting a crucial infrastructure for guiding future investigations into Mpox treatment. Additionally, Mpox-virus-induced immunological alterations and their potential immunopathogenesis should be further examined [14]. In this review, we focus on transmission routes, proposed pathophysiology, epidemiology, clinical diagnostics, phylogenetic clades of the Mpox virus and their evolutionary divergence, virology, vaccines, and treatment strategy for the Mpox virus in an update.

## 2. Transmission Route Associated with Mpox

**Figure 1:**
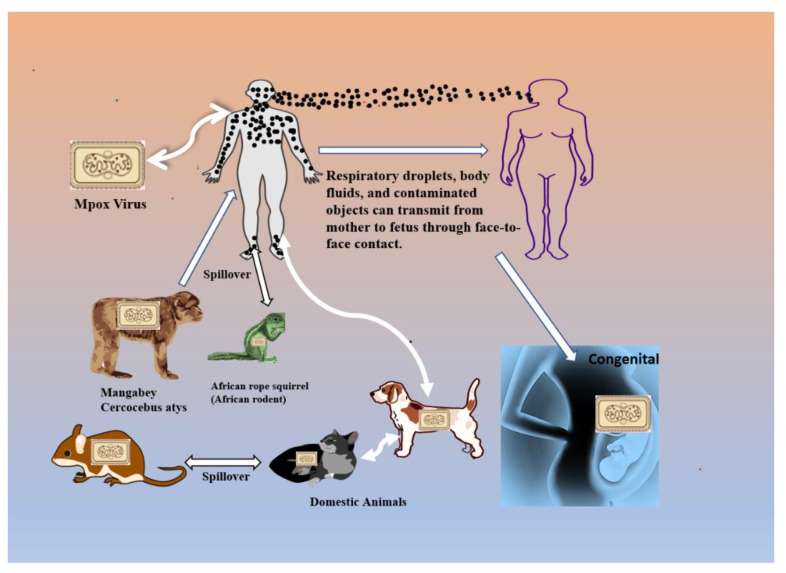
Transmission routes associated with Mpox-virus infection.

The current research implies that monkeypox may spread in three different ways: from person to person, via direct contact with infected organisms, and from animals to people. It is well established that animals can pass the Mpox virus on to humans [15]. The majority of the animals that are known to be carriers of the virus are rodents, such as rats, squirrels, and dormice, as well as numerous kinds of primates. On the other hand, there is evidence of a human-to-human transmission that has occurred not just in Africa but also outside the continent. Direct contact with skin lesions of infected animals or people, respiratory exposure to droplets from infected humans, and consumption of contaminated bushmeat are all potential routes of transmission for the Mpox virus [16,17]. During the current outbreak of the illness, researchers have shown that it is more prevalent in men who engage in sexual activity with other men [18]. Most cases of Mpox have been identified in men who have had sex with other men (MSM). The CDC reports that transmission can occur through contact with an infected person. Furthermore, semen analysis for many patients has revealed the presence of monkeypox-virus DNA, which is a novel finding [19]. The virus may pass from one person to another by respiratory (airborne) contact, direct contact with body fluids from an infected person, or during pregnancy from the mother to the fetus. Given that the pathogenic Mpox virus can be isolated from samples of semen, there are signs that transmission may happen during sexual intercourse [19,20,21]. The Mpox virus could be stored in the genital area if it stays in seminal fluids for a long time [11]. Whether the virus can spread via vaginal secretions is unknown. Even with adequate personal protective equipment, the virus may spread through fomites or by indirect contact with lesion material, such as through contaminated bedding, most commonly through inhalation [22]. Sharing a bed or room or using the same utensils as an infected individual are risk factors for transmission. Factors involving the introduction of the virus to the oral mucosa are linked to increased transmission risk. It is still unknown whether those who do not have monkeypox symptoms can transmit the virus [23]. Currently, further study is being conducted to better understand how this particular strain of the West African lineage spreads, although the general consensus is that it is not unique [24,25]. As far as we know, it does not disperse in the air like COVID-19. Mpox, in contrast to COVID-19, is not communicable until the infected individual develops symptoms. Therefore, it is much simpler to keep sick people apart and stop the spread of the disease. The transmission routes are explained in Figure 1 [26].

## 3. Proposed Pathophysiology of Mpox Virus

**Figure 2:**
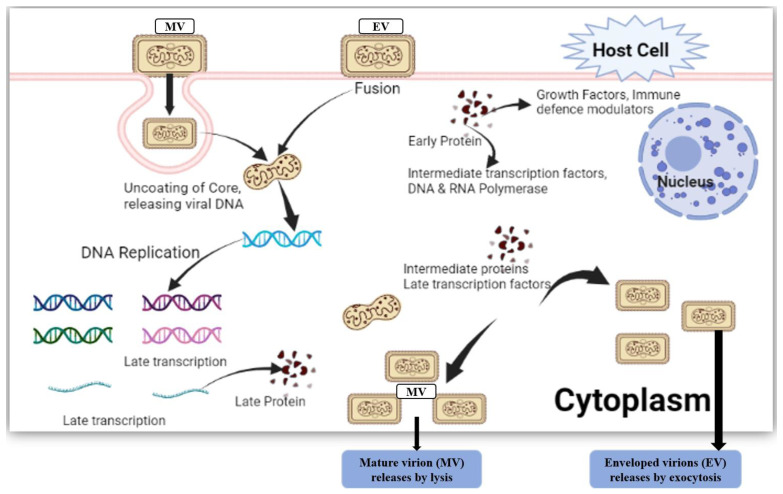
Cytosolic Mpox-virus pathways for the viral life cycle. The enveloped virion (EV) enters the host cell by fusion and the mature virion (MV) by micropinocytosis or fusion.

The Mpox virus is categorized as a category of the genus Orthopoxvirus under the family Poxviridae [27]. Macropinocytosis, endocytosis, and fusion are the three mechanisms through which poxviruses enter the cells of their hosts [28]. The DNA that makes up the Mpox-virus genome is linear and double-stranded (197 kb). The life cycle of the Mpox virus takes place in the cytoplasm despite the fact that it is a DNA virus. Replication of viral DNA, transcription of viral genes, and assembly of viral particles all need the presence of certain proteins [29]. The two primary forms of contagious virions generated by compromised cells are intracellular mature virus (IMV) and extracellular enveloped virus (EEV). These infectious virions are likely to be the most prevalent forms of the virus. EEVs are capable of moving quickly through an infected individual’s body to reach different areas, whereas IMVs are what spread the virus from one cell to another [30]. The Mpox virus has been divided into two separate genetic groups, West African (WA) and Congo Basin (CB), which is also called the Central African group [31]. The CB clade has been observed in areas ranging from Cameroon’s central and southern areas to the DRC, whereas the WA clade has been reported from Cameroon’s western areas to Sierra Leone [32]. It is commonly assumed that the WA clade is more likely to cause epidemics through spillover from animal hosts, whereas the CB clade is considered to be the most hazardous, as it is capable of sustained human-to-human transmission with intense secondary attack rates. Moreover, the WA clade produces the mildest symptoms, whereas the CB clade is seen as the most dangerous [30]. The Mpox-virus genome sequence of the current strains found in Europe (Portugal) fits the West African clade, according to recent sequencing data, indicating a milder version of the disease, although this has to be validated [33]. Additionally, the Mpox virus has three additional entry points into its host (human): the oropharynx, the nasopharynx, and intradermally. At the location of the vaccination, the virus multiplies before spreading to nearby lymph nodes. The virus spreads to other bodily organs after an initial phase of viremia. The Mpox virus resembles other recognized orthopoxviruses in terms of appearance. The Mpox virus has an exterior membrane made of lipoproteins and is oblong or brick-shaped [30]. The 2003 pandemic in the West African clade in the United States provides proof that the disease’s intensity may differ between clades. Humans and other primates are frequently less severely affected by West African monkeypox infections than animals [34,35]. Despite this, there were no fatalities reported during the epidemic that occurred in the United States in 2003 despite the fact that numerous people were hospitalized [36]. The Congo Mpox virus causes T-cell activation via the T-cell receptor (TCR). It is interesting to note, though, that when human cells are produced from people who have already contracted the monkeypox virus, the generation of inflammatory cytokines is suppressed. This shows that the Mpox virus may create a modulator that inhibits the responses of the host T cells [37]. The Central African clade has a complement-inhibiting gene, whereas the West African clade does not. It is an immune-modulating factor that may boost the Central African clade’s pathogenicity compared to that of the West African clade [38,39]. Apoptosis in the host may be precisely modulated, which suggests that the Central African monkeypox clade preferentially downregulates host responses in comparison to the West African clade [40]. Three West African strains (SL-V70, COP-58, and WRAIR-61) and one Central African strain (ZAI-96) were compared. The results showed a nucleotide difference of 0.55–0.56% between the Central African strains and the West African strains [41]. Two viral strains were discovered to have distinct clusters through genomic research. Whereas the West African strain is anticipated to contain 171 unique genes, the central African strain has 173 distinct functional genes. The two strains differ in their virulence; thus, 56 virulence genes were looked at, and 53 of them were present in both strains. The orthologs of BR-203, BR-209, and COP-C3L are where the two strains diverge most noticeably from one another [42]. Cytosolic Mpox-virus pathways for the viral life cycle are shown in Figure 2.

## 4. Epidemiology

**Figure 3:**
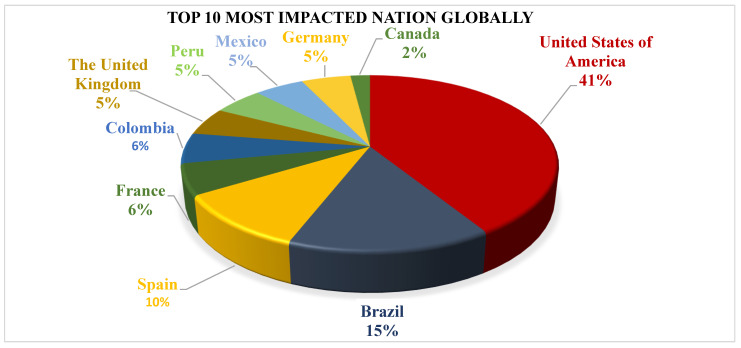
Top 10 most impacted nations globally are the United States of America (*n* = 29,860), Brazil (*n* = 10,709), Spain (*n* = 7518), France (*n* = 4114), Colombia (*n* = 4066), the United Kingdom (*n* = 3735), Peru (*n* = 3723), Mexico (*n* = 3696), Germany (*n* = 3690), and Canada (*n* = 1460).

The Mpox virus was first reported in 1958 in laboratory monkeys employed for research purposes at State Serum Institutes in Copenhagen, Denmark, as well as in Africa [18,43]. Humans in Sub-Saharan Africa have been infected with monkeypox through intimate contact with diseased animals, suggesting that the disease has been present for thousands of years. Mpox was formally recognized as a different illness in 1970, when the smallpox-eradication campaign revealed a continuing occurrence of smallpox-like disorders in rural regions [44,45]. Imported human Mpox-virus infections beyond the African continent have been infrequent in the last 50 years. Mpox has gained attention as a disease of global public-health significance since the first outbreak in the United States in 2003, which was linked to an infected pet prairie dog [46]^.^ It was believed that native prairie dogs housed alongside rats from Ghana introduced in Western Africa were the main source of the pandemic. This is because most infected individuals became ill after coming into contact with pet prairie dogs [45] In the summer of 2003, a cluster of illnesses in the US Midwest was attributed to Mpox. The main cause of the epidemic was believed to be native prairie dogs that were kept with rats imported from Ghana in Western Africa. This conclusion was reached as the vast majority of those who became infected fell ill after being in contact with pet prairie dogs [44]. Since 2003, many cases of Mpox have been reported in a variety of countries, with Nigeria experiencing the worst epidemic in 2017 [46]. In 2018, two individuals with secondary Mpox illness were reported by the United Kingdom after they visited Nigeria [47]. Over the past five years, there have been multiple cases of human Mpox identified in areas all over Africa [48,49]. Mpox has also spread to other areas, such as Singapore, Israel, the United States, and the UK [50,51,52]. On 7 May 2022, the UK Health Security Agency announced a confirmed case of Mpox in a person who had recently traveled to Nigeria [53]. By 29 January 2023, the World Health Organization had received 85,189 suspected and/or confirmed cases of Mpox from 110 countries, with the majority of cases occurring in Europe and the Americas, resulting in 86 fatalities around the world [54]. According to an epidemiological-modeling study, the Ro value for Mpox varies from 1.10 to 2.40 in countries with little exposure to Orthopoxvirus species. Ro is also known as the reproduction ratio, and it is used to determine the disease’s transmissibility [55]. This score indicates that an Mpox pandemic is poised to break out in the case of imported human or animal cases. As previously noted, the stated Ro indicates that each infected person has the ability to infect one to two other people. Because the virus is infectious, an infected individual must take special steps to isolate themselves and prevent contact with others [56,57]. Globally, the number of weekly reported new cases dropped by 2.3% in week 3 (16 January–22 January) (*n* = 295 cases) compared to week 2 (9 January–15 January) (*n* = 302 cases). The bulk of cases recorded in the last four weeks were from the Americas region (77.7%) and the African region (13.9%). The United States of America (*n* = 29,860), Brazil (*n* = 10,709), Spain (*n* = 7518), France (*n* = 4114), Colombia (*n* = 4066), the United Kingdom (*n* = 3735), Peru (*n* = 3723), Mexico (*n* = 3696), Germany (*n* = 3690), and Canada (*n* = 1460) are the ten most afflicted nations worldwide listed in Figure 3 [58]. These top affected nations account for 85.2% of all cases recorded worldwide. In the last seven days, 18 nations have reported an increase in the weekly number of cases, with Costa Rica reporting the largest rise. In the last 21 days, 74 nations have reported no new cases [54]. According to the CDC, India has had a total of 22 cases and 1 death due to the Mpox virus. Mpox showed up out of the blue in several countries and regions, but there was no initial epidemiological link to areas where the Mpox virus has always been common. This suggests that transmission has been going on for a long time without being noticed. Therefore, the monkeypox epidemic needs to be looked at with an open mind and with care. The World Health Organization (WHO) reports a moderate risk to the entire world. However, the risk is high in the Americas region and moderate in the Africa, Eastern Mediterranean, Europe, and South-East Asia regions, according to the WHO. In contrast, the risk is believed to be low in the Western Pacific region [58].

## 5. Clinical Symptoms and Diagnosis of Mpox Virus

Mpox is a virus belonging to the Orthopoxvirus genus and has a clinical presentation similar to smallpox. Its incubation period in humans typically ranges from four to 14 days but can be as long as 21 days. The disease begins with a febrile prodrome, which is accompanied by headache, muscle aches, backache, exhaustion, sweats, and fatigue. One to three days after the onset of fever, a rash appears on the face, inside the mouth, and on the hands, feet, chest, genitals, anus, and eyes. This rash begins as a flat macula and then becomes a papule before forming a vesicle filled with clear liquid. This clear liquid then turns into a yellowish liquid and forms pustules. Once the pustules, crusts, and lesions fall off, the patient is no longer considered infectious. However, scarring from the rash is a common outcome of an infection. More severe complications such as pulmonary distress, bronchopneumonia, ocular infections, corneal scarring, and even permanent damage, as well as lymphadenopathy, may also occur. It can be difficult for clinicians to differentiate Mpox from other viral or nonviral diseases due to its nonspecific clinical presentations. Therefore, laboratory diagnosis is imperative [59]. In order to accurately diagnose Mpox, health providers should collect an appropriate specimen and send it securely to a suitable laboratory. This is because verifying human Mpox virus depends on the type of sample and the available laboratory tests [60]. The symptoms of this disease are very hard to distinguish and difficult to manage in low-income countries, which is why it is a global issue, as these regions are seen as endemic with the disease [61]. The confirming processes for examining specimens and determining Mpox virus are genetic, phenotypic, and immunological methods [30]. Table 1 lists the types of monkeypox tests that can be implemented to identify human Mpox virus, and these strategies are more effective when combined with medical and epidemiological data, such as the patient’s immunization history [61,62].

## 6. Phylogenetic Clades of Mpox Virus and Their Evolutionary Divergence

Phylogenomic studies indicate that there are at least three distinct clades of the Mpox virus—Clade 1, associated with West Africa; Clade 2, connected to the Congo Basin; and a novel Clade 3, proposed following the 2022 European outbreak. Although the clinical appearance of smallpox and Mpox viruses are similar, the three clades suggest a distinct evolutionary divergence [63]. The Mpox virus is capable of infecting its host through various pathways, including the oropharynx, nasopharynx, and intradermal routes. It multiplies at the site of vaccination before eventually spreading to the lymph nodes. Once the initial viremia phase has passed, the virus proceeds to spread to other organs. The Mpox virus has an outer membrane composed of lipoproteins and is similar in appearance to other orthopoxviruses, typically presenting as an oblong or brick-shaped structure [30]. The Mpox virus requires specific proteins to be present for it to replicate its DNA, transcribe its genes, and assemble its viral particles. Its genome consists of linear, double-stranded DNA (197 kb), but the virus undergoes its life cycle in the cytoplasm, indicating its classification as a DNA virus [29]. There are three different ways in which poxviruses can enter the cells of their hosts: macropinocytosis, endocytosis, and fusion [28]. The Poxviridae family includes viruses with double-stranded DNA that can affect many creatures, such as birds, reptiles, insects, and mammals. It can be divided into two subsections: Chordopoxvirinae (with 18 genera and 52 species) and Entomopoxvarinae (consisting of 4 genera and 30 species). Monkeypox is part of the Poxviridae family, the Chordopoxvirinae subfamily, and the Orthopoxvirus genus [64,65]. The poxvirus species variola (smallpox), cowpox, monkeypox, vaccinia, camelpox, Alaskapox, Yaba monkey tumor virus, tanapox virus, orf virus, pseudocowpox virus, bovine papular stomatitis virus, buffalopox, and molluscum contagiosum have all been identified as causing illnesses in humans. Variola and molluscum contagiosum require humans as their main host [66]. The Mpox virus has been able to endure for quite a while in wild animals because of its huge range of potential hosts, and has sporadically spread to humans through spillover events [64]. The significant aspect of orthopoxviruses is that they demonstrate immunological cross-reactivity and cross-protection, meaning that being infected with any one of these viruses will provide some level of protection against any other member of the same genus [67,68]. Viruses with a genome of 200–500 kb and a brick-like shape can range in size from 140–450 nm and are known as orthopoxviruses [31,64,69]. There are more than 200 gene codes in the orthopoxvirus genome, and though some of them are not necessary for virus replication in cell culture, they may play an important role in the host’s antiviral defenses [70]. All poxviruses utilize intricate molecular pathways in order to complete their replication cycle in the cytoplasm of infected cells [70,71]. Substantial study has been conducted on the intracellular-replication cycle of the vaccinia virus, the vaccine developed from which helped eradicate smallpox worldwide. Other poxviruses share some of its important components [70,71]. Two distinct types of the virus, the internal mature virion and the external wrapped virion, which possess different surface glycoproteins, could potentially initiate the infection cycle. It is thought that glycosaminoglycans, which are found on the surfaces of mammalian cells, are necessary for the virus to attach itself to the cell membrane, even though not all cellular receptors have been determined [70,71]. It is believed that millions of people worldwide perished due to smallpox [72]. Smallpox is widely regarded as one of the most feared infectious diseases in human history. The consequences of the disease are a reminder of the destructive capacity of orthopoxviruses. Despite the lack of clarity around the origins of smallpox, there is some evidence to suggest that the variola virus may have descended from an ancient rodent poxvirus [72]. People have been aware for a long time of the danger of zoonotic orthopoxvirus diseases such as the Mpox virus to both humans and other animals [73,74,75]. Due to the suspension of smallpox-immunization programs over 40 years ago, a large portion of the global population has no immunity to smallpox and zoonotic orthopoxviruses. This raises the possibility that a zoonotic orthopoxvirus like the Mpox virus may gain the ability to spread more easily among humans if certain conditions are met, such as an increase in human infections and the continued absence of vaccination protection [73]. Researchers must focus on the virology of how pox viruses are altering normal cells and damaging organs.

## 7. Vaccines for Mpox Virus

In order to prevent the spread of the Mpox virus and protect people from it, there are multiple steps that must be taken. Vaccines are the most effective way to achieve this goal, yet unfortunately, there is currently no specific vaccine for the Mpox virus. However, research has revealed that the same smallpox vaccine that was used to protect people against smallpox may be effective in protecting against the Mpox virus as well [76]. Previous knowledge shows that receiving a smallpox vaccination could result in a reaction to the Mpox virus and may be able to protect from being infected by the virus by up to 85% [41]. The Food and Drug Administration has approved ACAM2000, a second-generation smallpox vaccine, to be used to prevent exposure to smallpox during an outbreak or crisis. Therefore, it has been acquired for the Strategic National Stockpile (SNS) and is available to be used for a range of demographic groups [77]. Furthermore, JYNNEOS (MVA-BN) was authorized to be used in the United States and Canada in 2019 after a series of animal studies. Clinical trials have also demonstrated its strong effectiveness and safety, which can be used to protect people in many different age groups from getting infected with the Mpox virus [78,79,80]. The approval was due to the effectiveness in animals, the safety profile in humans, and the evidence that JYNNEOS had a similar immunogenicity to existing smallpox vaccinations [81,82,83]. In addition, due to the verified protective effects in animal studies and the immunizing action seen in human trials, the US Food and Drug Administration’s emergency investigational new drug program approved LC16 both in the US and in Japan [84,85]. No data exists on the efficacy of LC16 for avoiding Mpox-virus infections, even though it is the only smallpox vaccine available for kids. It is essential to note that when taking pre-exposure precautions, these vaccinations can often stop Mpox-virus infection. Yet, experts have demonstrated that post-exposure immunization may be able to stop the onset of serious diseases or reduce the intensity of the issues experienced by those who have been infected with the Mpox virus [86]. In this scenario, it is recommended to get vaccinated promptly after being exposed. The Centers for Disease Control and Prevention (CDC) has confirmed that vaccination within four days of exposure can prevent the onset of illness. If this window is missed, the disease may still occur, but immunization in the first two weeks can help to avoid more serious consequences [87]. Currently, three vaccines for orthopoxviruses are accessible: ACAM2000, JYNNEOS, and LC16. The initial vaccine, ACAM2000, is a replicating vaccine; however, the other two vaccines are either non-replicating or minimally replicating. In 2015, the Food and Drug Administration (FDA) and the United States government gave ACAM2000 a permit to treat smallpox and monkeypox. From 2015 to 2019, it was the only monkeypox vaccine that could be purchased in the United States [86,88]. Cell-culture techniques were utilized in both France and the USA to create ACAM2000. Those aged from 18 to 64 were allowed to use it. Emergent BioSolutions manufactured this second-generation, replication-capable, live-attenuated, plaque-purified vaccine. The scarification technique with a bifurcated needle is used to deliver the vaccine percutaneously by repeatedly injecting it into the surface of the skin. This single-dose vaccine grants maximum immunity 28 days after immunization. People exposed to highly virulent orthopoxviruses require booster doses every three years, whereas those exposed to low-virulent orthopoxviruses (e.g., vaccinia virus or cowpox virus) must be administered booster doses every 10 years [88]. The MVA-BN vaccine created by Bavarian Nordic is a third-generation, live-attenuated, nonreplicating Ankara vaccine. It is a two-dose vaccination that must be taken 28 days apart in order to generate immunity. Clinical trials indicated that a substantial antibody response was seen after the initial dosage. Following the second dose, immunity was established. Those exposed to highly virulent orthopoxviruses need to receive a booster shot every two years, whereas those exposed to low-virulence strains need one every 10 years. In 2019, this vaccine was given the green light by the FDA for use in Canada to prevent smallpox and monkeypox in adults aged 18 and older who are at high risk [89,90,91]. KM Biologics created a third-generation vaccination called the LC16 vaccine, which was granted a license for use against smallpox in Japan in 1975 and against monkeypox in the USA in 2014 [92]. This live-attenuated, minimally replicating vaccine was made using cell-culture techniques and has an immunogenic-membrane protein B5R that has been eliminated [85]. A bifurcated needle is used to percutaneously administer the multidose vaccination, which is suitable for individuals of all ages, including newborns and toddlers [92]. It is essential to look into the reactogenicity, safety, and any possible adverse effects of the vaccine in order to ensure the most effective selection, especially for those in high-risk and vulnerable groups. For example, there are various vaccines that can be used for healthy individuals, such as those that are nonreplicating (e.g., JYNNEOS), slightly replicating (e.g., LC16), and replicating (e.g., ACAM2000) [82,83]. Researchers need to make a vaccine specifically for the monkey-pox virus to increase protection against the virus.

## 8. Treatment for Mpox

**Figure 4:**
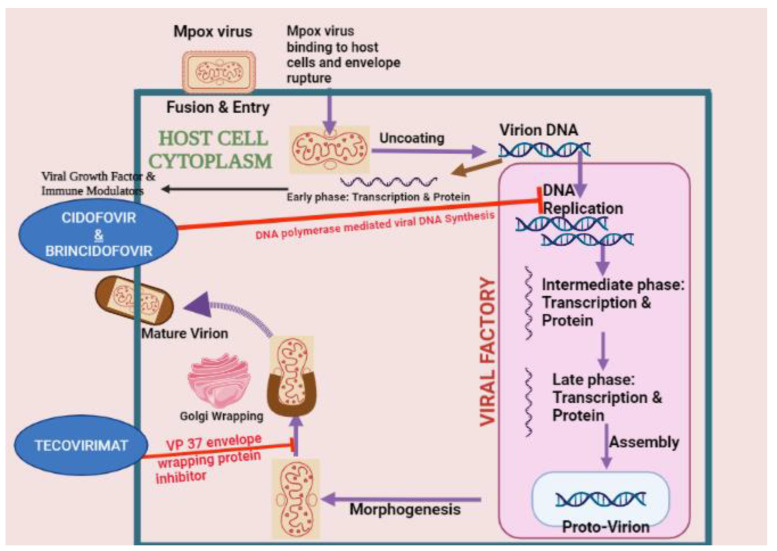
An illustration of the life cycle of the Mpox virus inside the host-cell cytoplasm to elicit the mechanism of action of three different antiviral therapies: cidofovir, brincidofovir, and tecovirimat.

A total of 85,189 cases of Mpox infection as of 29 January 2023 have alarmed the world. Historically, immunization against the vaccinia virus could safeguard against Mpox; however, since smallpox was eradicated, this type of vaccination has ceased. Consequently, therapeutic options for those already infected are of considerable importance [93]. No antiviral drugs that have been approved by the American Food and Drug Administration are specifically created to target the Mpox virus. However, other medications such as tecovirimat (TPOXX/ST-246) and brincidofovir, both of which are effective against smallpox, as well as cidofovir, an antiviral approved to fight CMV, have been shown to be effective against orthopoxviruses in laboratory experiments. In 2018, the US Food and Drug Administration (FDA) approved tecovirimat (TPOXX) for the treatment of smallpox in adults and children. This medication works by preventing VP37, a viral-envelope-wrapping protein, from functioning properly and blocking viral replication and release. It is currently available in the US free of charge under an expanded-access investigational new drug protocol (EA-IND) [94]. Tecovirimat can be taken orally or intravenously. Although there is not enough evidence yet to show how effective it is for treating Mpox, it has been reported to have mild side effects like headache, nausea, vomiting, abdominal pain, and neutropenia in one trial participant [95]. The use of an intravenous formulation may lead to redness, pain, and swelling at the area of infusion [94]. In June 2021, the FDA approved the use of brincidofovir against smallpox in both adults and children. This prodrug of cidofovir comprises a lipid conjugate and is converted to cidofovir diphosphate (CDP) within the cells, which inhibits the viral DNA polymerase, eventually stopping the replication of the virus. Although there is a dearth of data on the use of brincidofovir against MPXV, animal studies have revealed that when treatment was administered at the appropriate time, infected prairie dogs had survival rates of between 29 and 57% [96]. Adler et al. reported three cases of human Mpox that were addressed by administering brincidofovir. However, the treatment was discontinued due to a rise in the levels of liver enzymes. An advantage of brincidofovir over cidofovir is that it is available in both pill and liquid forms and is smoother on the kidneys [96]. Cidofovir and its prodrug, brincidofovir, have the same method of working. There is a lack of evidence from humans on the effectiveness of cidofovir against monkeypox, but there are animal studies that show that it is useful against orthopoxviruses such as cowpox, vaccinia, ectromelia, and rabbitpox [97]. Thornhill et al. mentioned cases from the 2022 Mpox outbreak being treated with cidofovir, which is only obtainable as an intravenous formulation but can carry a risk of severe renal toxicity [98]. In the treatment of the Mpox virus there is no specific treatment available in the current outbreak, so researchers should work on a treatment strategy. In Figure 4 below, the life cycle of Mpox virus inside the host-cell cytoplasm is illustrated to elicit the mechanism of action of three different antiviral therapies: cidofovir, brincidofovir, and tecovirimat [99].

## 9. Challenges

Currently, operational research faces challenges in understanding the dynamics of monkeypox transmission and control due to limited resources for detailed case investigations and contact follow-up in affected communities. A serious issue is the lack of adequate diagnostic facilities in laboratories. The difficulty in diagnosing the Mpox virus arises from the insufficiency of laboratory-diagnosis capacity and access, making it challenging to identify any underlying etiology. To comprehend the epidemiology and subclinical infection among contacts in communities, a seroprevalence study is crucial. However, currently available serological assays are generic orthopox tests, and they cannot specifically identify the Mpox virus due to cross-reactivity between the Mpox and smallpox viruses. Hence, it is challenging to distinguish between Mpox-virus infection and prior smallpox vaccinations or other orthopoxvirus infections. Moreover, these assays are not available on the marketplace. Data collected from Nigeria reveal that approximately 20% of 70 monkeypox-negative patients with a rash illness that had similar antigens also had orthopox antibodies. In addition, there is no specific antiviral treatment for Mpox. Treatment is primarily supportive, focusing on managing symptoms such as fever and rash. Vaccination with the smallpox vaccine can provide some protection against Mpox, but the vaccine is not widely available in many countries. Further research, including molecular and genomic approaches, is necessary to identify other orthopoxviruses transmitted in human and animal populations.

## 10. Conclusions and Future Prospective

Mpox is a viral disease that is closely related to smallpox and primarily found in remote parts of Central and West Africa. There is currently no specific vaccine for Mpox, but the smallpox vaccine provides some protection against the disease. However, the smallpox vaccine is no longer routinely administered, and many younger people in Africa may not have received it. Several vaccines are being studied for their potential effectiveness against Mpox, including live-attenuated Mpox vaccines, DNA vaccines, and recombinant vaccines. Synthetic peptide-based prototype vaccines have also shown promise in preclinical studies, and researchers are investigating the use of mRNA vaccines for booster purposes in those who have received the mRNA vaccine for COVID-19. Overall, the development of effective vaccines for Mpox is an ongoing area of research, and scientists are working to develop new vaccines that can protect against this rare but potentially serious disease.

## Figures and Tables

**Table 1:** Types of diagnostic tests for Mpox.

Scheme	Types of Mpox Tests	Description	Specimen Taken
1.	Polymerase chain reaction (PCR)	In nucleic-acid-amplification testing, also known as molecular testing or PCR, the laboratory technician extracts genetic material from a patient specimen and subsequently amplifies it using pathogen-specific primers. Upon amplification, if the virus is present in the sample, the test detects it, thereby revealing whether the patient is actively infected at the time of testing. PCR is the preferred laboratory test for monkeypox diagnosis due to its high sensitivity and accuracy.	Lesion biopsy
2.	Viral culture	Routine diagnostic procedures do not include virus isolation, and it should only be conducted in laboratories that possess adequate expertise and containment facilities. Virus isolation is not a standard diagnostic approach.	Lesion fluid
3.	Electron microscopy	In evaluating the sample for a potential poxvirus, electron microscopy is an option, but due to the high technical skills and facility required and the availability of molecular assays, this method is not routinely used for the diagnosis of poxviruses.	Biopsy specimen, scab material, vesicular fluid
4.	Immunohistochemistry	A check for orthopoxvirus-specific antigens is done through testing.	Biopsy specimen
5.	Anti-Orthopoxvirus IgG and IgM tests	These tests can be utilized to assess either recent or past exposure to orthopoxvirus.	Blood specimen

Abbreviations: DNA, deoxyribonucleic acid; IgM, immunoglobulin IgG, immunoglobulin G.

## Data Availability

This section provides details regarding where data supporting reported results can be found, including links to publicly archived datasets analyzed or generated during the study.
